# The Association between Obesity and Cancer Risk: A Meta-Analysis of Observational Studies from 1985 to 2011

**DOI:** 10.5402/2013/680536

**Published:** 2013-04-04

**Authors:** M. Dobbins, K. Decorby, B. C. K. Choi

**Affiliations:** ^1^School of Nursing, McMaster University, 1280 Main Street West, 3N25G, Hamilton, ON, Canada L8N 3Z5; ^2^Department of Epidemiology and Community Medicine, University of Ottawa, Ottawa, ON, Canada K1H 8M5

## Abstract

*Background*. Cancer and cardiovascular diseases are the leading causes of mortality and morbidity worldwide. The purpose of this meta-analysis is to synthesize the evidence evaluating the association between obesity and 13 cancers shown previously to be significantly associated with obesity. *Methods*. Relevant papers from a previously conducted review were included in this paper. In addition, database searches of Medline and Embase identified studies published from the date of the search conducted for the previous review (January, 2007) until May, 2011. The reference lists of relevant studies and systematic reviews were screened to identify additional studies. Relevance assessment, quality assessment, and data extraction for each study were conducted by two reviewers independently. Meta-analysis was performed for men and women separately using DerSimonian and Laird's random effects model. *Results*. A total of 98 studies conducted in 18 countries from 1985 to 2011 were included. Data extraction was completed on the 57 studies judged to be of strong and moderate methodological quality. Results illustrated that obese men were at higher risk for developing colon (Risk Ratio (RR), 1.57), renal (1.57), gallbladder (1.47), pancreatic (1.36), and malignant melanoma cancers (1.26). Obese women were at higher risk for esophageal adenocarcinoma (2.04), endometrial (1.85), gallbladder (1.82), renal (1.72), pancreatic (1.34), leukemia (1.32), postmenopausal breast (1.25), and colon cancers (1.19). *Conclusions*. The results of this meta-analysis illustrate a significant, positive, and, for some cancers, strong association between obesity and cancer incidence. Given that approximately 23% of Canadians are obese, a significant proportion of cancer in Canada could be avoided if obesity was eliminated or significantly reduced.

## 1. Introduction

Chronic diseases are the leading cause of mortality and morbidity and contribute significantly to the overall health expenditures from both a societal perspective as well as an individual one [1]. Common chronic diseases include heart disease, stroke, cancer, emphysema, diabetes, and osteoporosis. Furthermore, cancer and cardiovascular diseases are the leading causes of mortality and morbidity worldwide [2], with cancer expected to result in 75,000 deaths per year in Canada [3] and 571,950 in the US [4].

While the leading risk factor for cancer continues to be tobacco use, evidence shows that obese men and women have a greater likelihood of developing and dying from cancer than those who are not [2, 5–7]. Obesity is defined as a Body Mass Index (BMI) of 30 kg/m^2^ or greater. Approximately a quarter of Canadian men and women are considered obese [9]. There are many contributing factors to obesity such as physical inactivity, unhealthy diet, genetics, and others such as metabolic, environmental, social, economic, and psychological factors. It is estimated that globally, every year, three to four million cases of cancer could be prevented by eating healthier and being more physically active [2]. 

With respect to morbidity, research demonstrates that obesity increases the risk of cancers of the esophagus, breast (postmenopausal), endometrium, colon and rectum, kidney, pancreas, thyroid, gallbladder, and possibly other cancers as well [8]. Other evidence suggests that obesity leads to an increased risk for thirteen cancers including esophageal adenocarcinoma, thyroid, colon, rectal, renal, endometrial, pancreatic, gallbladder, postmenopausal breast, malignant melanoma, multiple myeloma, leukemia, and non-Hodgkin lymphoma [6]. The cost of obesity to the health care system in Canada is estimated to be 4.3 billion per year [10]. Elsewhere the cost of obesity to the health care system has been found to represent 2.3% of annual hospital care costs [11]. Physical inactivity, which contributes to obesity, has been associated with significant health care expenditures for numerous chronic diseases including cancer. Canadian research estimates the economic burden of obesity as $2.1 billion in both direct and indirect costs [12]. Data from that same study suggests that a 10% decrease in inactivity would result in health savings of $150 million per annum [13]. 

The purpose of this meta-analysis is to synthesize the evidence evaluating the association between obesity and cancer. Specifically the association between obesity and thirteen cancers shown previously [6] to be significantly associated with obesity is the focus of this meta-analysis. In addition, among cancers for which a statistically significant positive association with obesity is observed, population-attributable risk for the Canadian population is calculated and reported. 

## 2. Methods

### 2.1. Search Strategy

**Figure 1:**
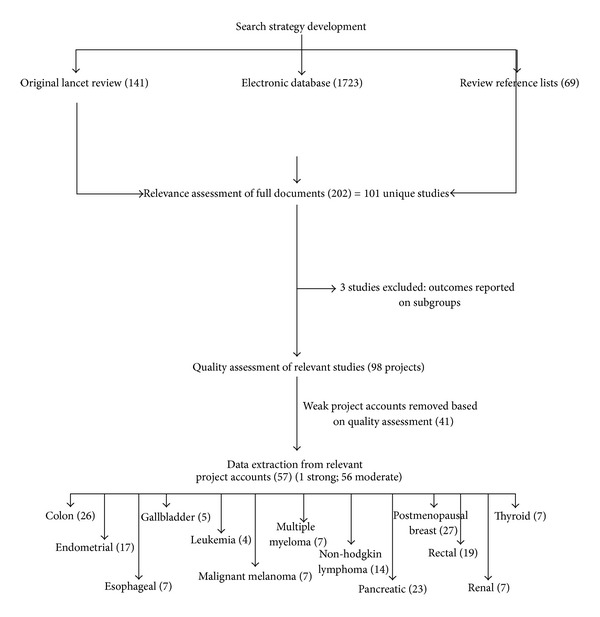
Overview of review process.

Several activities were included in the search strategy to identify primary studies for this paper. An overview of the review process is depicted in Figure 1. First, the primary studies included in Renehan et al., for which significant associations between obesity and cancer were reported, were identified and retrieved in full document version. The search strategy employed by Renehan et al. identified studies published between 1985 and 2007, indexed in Medline and Embase. Three additional studies, noted by Renehan et al., but published after their search was conducted, and therefore not included in their review, were also retrieved. 

Second, a search for studies, conducted since the Renehan et al. search for primary studies was completed, was conducted using the following search strategy. The electronic searches performed by Renehan et al. were replicated from January 2007 to May 2011 in Medline and Embase through OVID. Titles and abstracts were screened for relevance by two independent reviewers. All references chosen by one or both of the reviewers as being potentially relevant were selected for further review and imported into Systematic Review Software (SRS) from the Centre for Evidence-Based Medicine. All potentially relevant studies were retrieved in PDF version. Finally, the reference lists of relevant studies were screened to identify additional potentially relevant studies, as well as the reference lists of published systematic reviews or meta-analyses on this topic. 

### 2.2. Relevance Assessment

Two reviewers independently screened all retrieved articles using an existing relevance assessment tool. The following four criteria were used to assess relevance to the research question: (1) is the article a primary study; (2) is the focus of the study to explore the relationship between obesity and cancer incidence in adults aged 18 years and older; (3) does the study report on any one or more of the following 13 cancers: esophageal adenocarcinoma, thyroid, colon, renal, endometrial, gallbladder, rectal, malignant melanoma, postmenopausal breast, pancreatic, leukemia, multiple myeloma, and non-Hodgkin lymphoma; (4) is data on the risk ratio or odds ratio between obesity and incidence of any one or more of the 13 cancers in adults aged 18 years and over provided. Both reviewers independently assessed each study for relevance and met to resolve discrepancies through discussion. All articles referring to the same study were considered as one study. 

### 2.3. Methodological Quality Assessment

All studies judged to be relevant were assessed for methodological quality by two independent reviewers using an existing quality assessment tool, based on the work of the Evidence-based Medicine group at McMaster University. The assessment criteria consisted of the following components: (1) research design; (2) identification of comparison groups, (3) comparison groups compared on important confounders at baseline, (4) outcomes and exposures measured in the same way in all groups being compared, (5) data collection tools shown to be valid, (6) data collection tools shown to be reliable, (7) follow up sufficiently long for the outcome(s) of interest; (8) completeness of followup, (9) temporality (exposure is known to precede outcome), (10) dose-response gradient, (11) significant baseline differences controlled for in the analysis, (12) appropriate statistical tests for the research design, (13) precision of estimate of effect, and (14) sufficient detail describing study participants.

Points were assigned to each criterion according to an a priori scale. Studies were given an overall score out of 20 possible points and were then classified into three categories: strong, moderate, and weak. Studies receiving an overall rating of 16 or more points were rated as strong. Those obtaining a score of 11–15 points received a rating of moderate, and those obtaining a score of 10 or less were rated as weak. Reviewers independently rated each study and met to resolve discrepancies in overall ratings through discussion. Studies deemed as being of weak methodological quality were excluded from further analysis as the validity of the results was questionable given the many limitations inherent in these studies. 

### 2.4. Data Extraction

Data on the population, study methods, and outcomes were extracted for each study independently by two reviewers. Discrepancies were resolved through discussion. 

### 2.5. Data Analysis

Estimates of association were measured as a risk ratio and meta-analysis performed as a weighted average of the log risk ratios and 95% confidence intervals. In instances where odds ratios were reported for primary studies (e.g., case-control studies) these were first converted into risk ratios as suggested by Renehan et al., and then the log risk ratios and coinciding 95% confidence intervals were calculated.

Tests of heterogeneity were conducted among studies using a Chi square procedure, where *P* < 0.05 was considered an indication of heterogeneity. Risk ratios were pooled using the DerSimonian and Laird random effects model, given that there was considerable variation in how independent and dependent variables were measured across studies, and because in most cases statistically significant heterogeneity across study results was observed. Risk ratios/odds ratios and 95% confidence intervals extracted from each primary study, where those that adjusted for the greatest number of confounding variables including behavioural factors. Analyses were performed and reported separately by sex. In all instances reference body mass index (BMI) was 18.5–24.99 which was compared to the BMI category of 30 or more. 

### 2.6. Population-Attributable Risk

Population-attributable risk (PAR) is the portion of the incidence of a disease in the population that is due to exposure. PAR% is the percent of the incidence of a disease in the population that is due to exposure. It is the percent of the incidence of the disease in the population that would be eliminated if exposure was eliminated. PAR% = [P(RR − 1)]/[1 + P(RR − 1)], where, P is the population prevalence of obesity, and RR is the pooled risk ratio [12]. The most current obesity rates for men and women available in Canada [9] were used to represent population prevalence. The same formula was used to compute the 95% confidence interval of PAR%.

## 3. Results

### 3.1. Search Results

In total 141 articles were identified from Renehan et al., 1723 from the database searches and 69 from reference lists; the total is 1933 papers. Of the 141 articles included in Renehan et al., 94 articles were relevant to the 13 cancers included in this paper. The remaining 47 articles from Renehan et al's review explored the association between obesity and cancers other than the thirteen of interest in this paper, and therefore were excluded. Of the 1723 articles identified in the database searches, 75 were judged to be relevant. Finally, of the 69 articles identified from the reference lists of relevant studies, 33 were deemed relevant. A total of 202 articles were deemed relevant for this paper. When papers were grouped according to independent studies, a total of 101 unique studies were relevant. The Kappa score for agreement between reviewers on relevance assessment was 0.835, indicating high agreement. Reasons studies found to be not relevant were data not reported on the 13 cancers, and/or the association between obesity and cancer in adults aged 18 years and older was not the focus of the study.

### 3.2. Methodological Quality Assessment

One hundred and one studies were assessed for methodological quality. It was identified during quality assessment that three studies (judged to be of moderate quality) reported data in a way that was inconsistent with the other studies, therefore could not be aggregated with other studies. These studies were excluded from the meta-analysis. Of the remaining 98 studies one was assessed as being of strong methodological quality, 56 were rated as moderate and 41 as weak. Data was extracted on the 57 strong and moderate studies. The following criteria distinguished strong and moderate studies from weak studies: strong and moderate studies tended to use measurement tools with proven validity and reliability, demonstrated that obesity preceded cancer incidence, and established a dose-response gradient. Studies of weak methodological quality rated poorly on these criteria as well as research design. A summary of the quality assessment of the 57 studies included in the meta-analysis is presented in Figure 1. Quality assessment of the weak studies not included in this publication can be requested from the primary author.

### 3.3. Study Characteristics

The study designs included 43 cohort and 14 case-control studies published between 1985 and 2011. The majority of studies were conducted in the United States (19), followed by Sweden (7), Norway (4), and Japan (5). Most studies had follow-up rates of 80% or greater. Education level ranged from primary school to postsecondary. Among the cohort studies participants were followed up between 6 and 39 years. 

### 3.4. Association between Obesity and Cancer Incidence

#### 3.4.1. Colon Cancer

**Figure 2:**
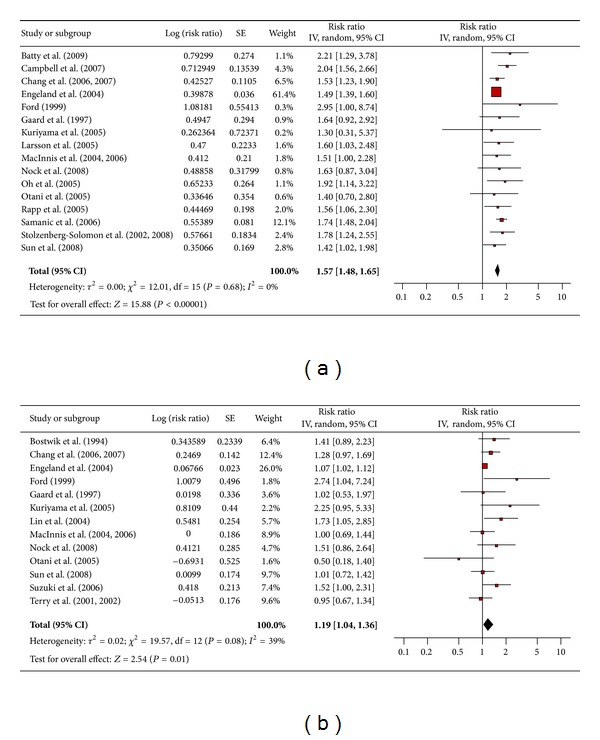
(a) Obesity and colon cancer in men. (b) Obesity and colon cancer in women.

Sixteen studies were included in the meta-analysis assessing the association between obesity and colon cancer among men (Figure 2(a)) and 13 studies among women (Figure 2(b)). The pooled risk ratio illustrated that obese men had an increased risk of colon cancer compared to men of normal weight (RR 1.57; 95% confidence interval 1.48 to 1.65). There was no significant heterogeneity across studies (heterogeneity *P* = 0.68). The pooled risk ratio illustrated that obese women had an increased risk of colon cancer compared to women of normal weight (RR 1.19; 95% confidence interval 1.04 to 1.36). There was significant heterogeneity observed across studies (heterogeneity *P* = 0.08). 

#### 3.4.2. Endometrial Cancer

**Figure 3:**
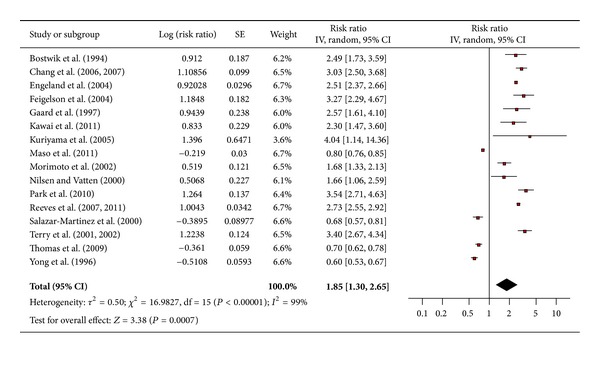
Obesity and endometrial cancer.

The pooled risk ratio from 16 studies illustrated that obese women had an increased risk of endometrial cancer compared to women of normal weight (RR 1.85; 95% confidence interval 1.3 to 2.65) (Figure 3). There was significant heterogeneity across studies (heterogeneity *P* = 0.00001). 

#### 3.4.3. Esophageal Adenocarcinoma

**Figure 4:**
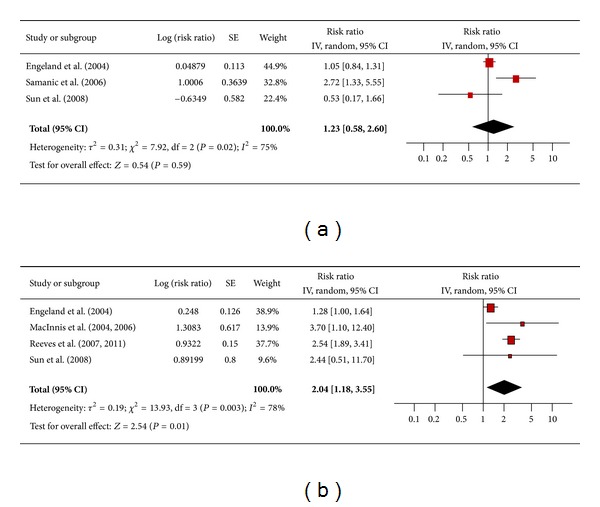
(a) Obesity and esophageal adenocarcinoma in men. (b) Obesity and esophageal adenocarcinoma in women.

There were 3 studies that assessed the association between obesity and esophageal adenocarcinoma in men (Figure 4(a)) and 4 studies among women (Figure 4(b)). The pooled risk ratio demonstrated that obese men did not have an increased risk for esophageal adenocarcinoma compared to men of normal weight (RR 1.23; 95% confidence interval 0.58 to 2.60). There was significant heterogeneity across studies (heterogeneity *P* = 0.02). Among obese women, however, the pooled risk ratio indicated a sizable increased risk of esophageal adenocarcinoma compared to women of normal weight (RR 2.04; 95% confidence interval 1.18 to 3.55). There was significant heterogeneity across studies (heterogeneity *P* = 0.003).

#### 3.4.4. Gallbladder Cancer

**Figure 5:**
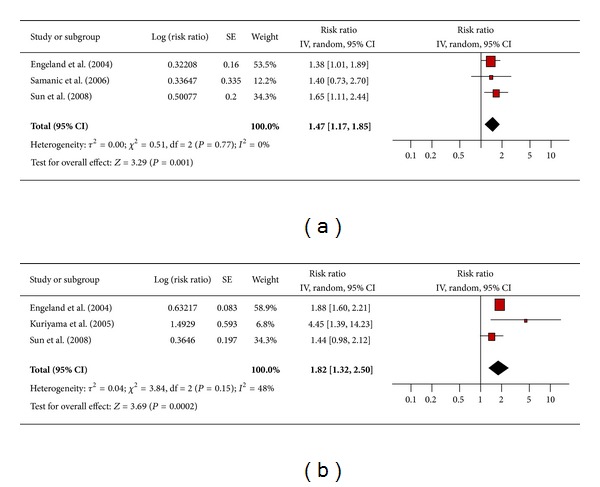
(a) Obesity and gallbladder cancer in men. (b) Obesity and gallbladder cancer in women.

There were 3 studies each assessing the association between obesity and gallbladder cancer in men (Figure 5(a)) and women (Figure 5(b)). The pooled risk ratio illustrated that obese men had an increased risk of gallbladder cancer compared to normal weight men (RR 1.47; 95% confidence interval 1.17 to 1.85). There was no significant heterogeneity across studies (heterogeneity *P* = 0.77). Similarly, among obese women the pooled risk ratio demonstrated an increased risk of gallbladder cancer compared to women of normal weight (RR 1.82; 95% confidence interval 1.32 to 2.50). There was no significant heterogeneity across studies (heterogeneity *P* = 0.15). 

#### 3.4.5. Leukemia

**Figure 6:**
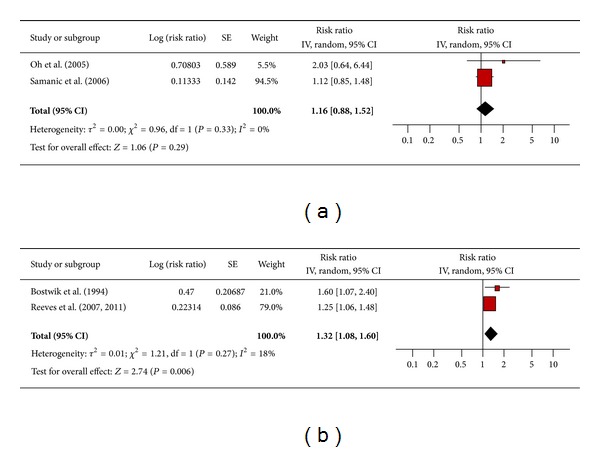
(a) Obesity and leukemia in men. (b) Obesity and leukemia in women.

There were 2 studies that assessed the association between obesity and leukemia among men (Figure 6(a)) and women (Figure 6(b)). The pooled risk ratio illustrated that obese men had no increased risk of leukemia compared to men of normal weight (RR 1.16; 95% confidence interval 0.88 to 1.52). For obese women, however, the pooled risk ratio illustrated an increased risk of leukemia compared to women of normal weight (RR 1.32; 95% confidence interval 1.08 to 1.60). There was no significant heterogeneity across studies for either men or women. 

#### 3.4.6. Malignant Melanoma

**Figure 7:**
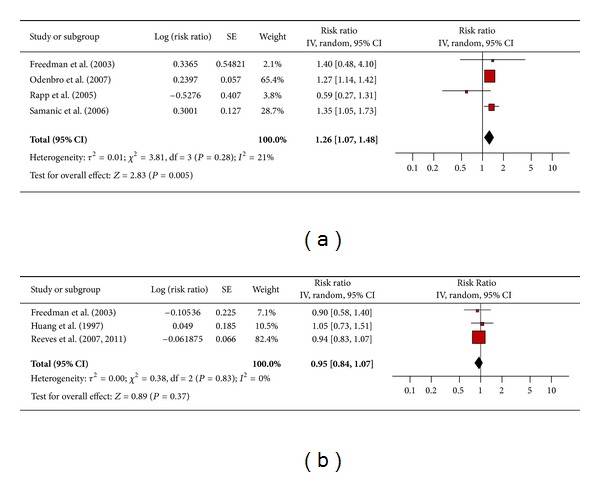
(a) Obesity and malignant melanoma in men. (b) Obesity and malignant melanoma in women.

Four studies assessed the association between obesity and malignant melanoma among men (Figure 7(a)) and 3 among women (Figure 7(b)). The pooled risk ratio illustrated that obese men had an increased risk of malignant melanoma compared to men of normal weight (RR 1.26; 95% confidence interval 1.07 to 1.48). There was no significant heterogeneity across studies (heterogeneity *P* = 0.28). The pooled risk ratio for obese women showed no increased risk of malignant melanoma compared to women of normal weight (RR 0.95; 95% confidence interval 0.84 to 1.07). There was no significant heterogeneity across studies (heterogeneity *P* = 0.83).

#### 3.4.7. Multiple Myeloma

**Figure 8:**
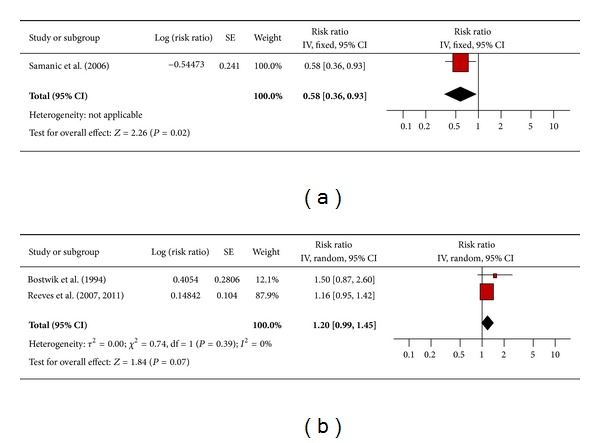
(a) Obesity and multiple myeloma in men. (b) Obesity and multiple myeloma in women.

Only one study for men (Figure 8(a)) and two studies for women (Figure 8(b)) assessed the association between obesity and multiple myeloma. The results reported by Samanic et al., 2006 showed obese men had significantly lower risk of multiple myeloma compared to men of normal weight (RR 0.58; 95% confidence interval 0.36 to 0.93). The pooled ratio for women showed an increased risk of multiple myeloma compared to women of normal weight, although the 95% confidence interval was just short of reaching statistical significance (RR 1.20; 95% confidence interval 0.99 to 1.45). There was no significant heterogeneity across the two studies (heterogeneity *P* = 0.39). 

#### 3.4.8. Non-Hodgkin Lymphoma

**Figure 9:**
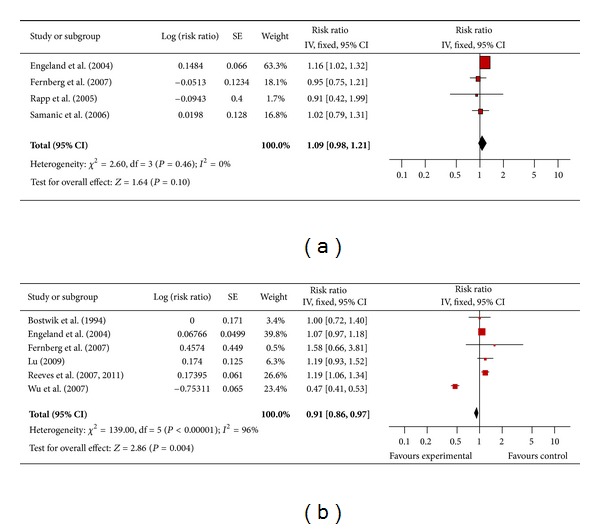
(a) Obesity and non-Hodgkin lymphoma in men. (b) Obesity and non-Hodgkin lymphoma in women.

Four studies in men (Figure 9(a)) and six in women (Figure 9(b)) assessed the association between obesity and non-Hodgkin lymphoma. The pooled risk ratio indicated there was no increased risk of non-Hodgkin lymphoma among obese men compared to men of normal weight (RR 1.09; 95% confidence interval 0.98 to 1.21). There was no significant heterogeneity across studies (heterogeneity *P* = 0.46). Among obese women, however, the pooled risk ratio showed a reduced risk of non-Hodgkin lymphoma compared to women of normal weight (RR 0.91; 95% confidence interval 0.86 to 0.97). There was significant heterogeneity across studies (heterogeneity *P* = 0.0001). 

#### 3.4.9. Pancreatic Cancer

**Figure 10:**
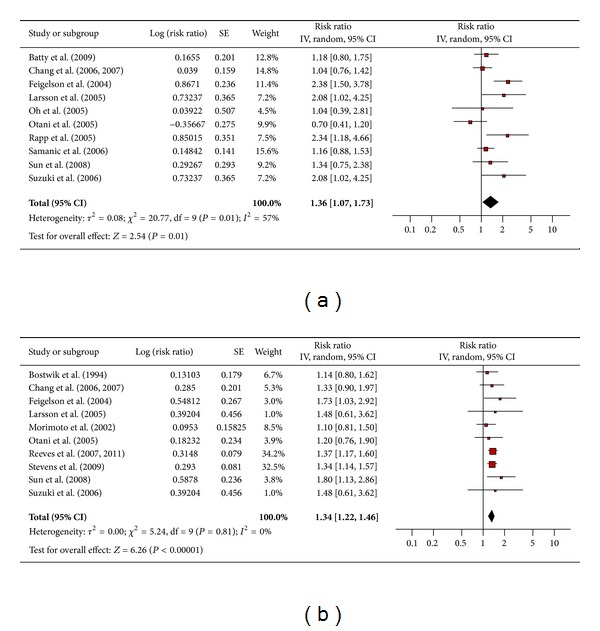
(a) Obesity and pancreatic cancer in men. (b) Obesity and pancreatic cancer in women.

Nine and ten studies, respectively, assessed the association between obesity and pancreatic cancer in men (Figure 10(a)) and women (Figure 10(b)). The pooled risk ratio illustrated that obese men had an increased risk of pancreatic cancer compared to men of normal weight (RR 1.36; 95% confidence interval 1.07 to 1.73). There was significant heterogeneity across studies (heterogeneity *P* = 0.01). Among obese women the pooled risk ratio also showed an increased risk of pancreatic cancer compared to women of normal weight (RR 1.34; 95% confidence interval 1.22 to 1.46). There was no significant heterogeneity across studies (heterogeneity *P* = 0.81). 

#### 3.4.10. Postmenopausal Breast Cancer

**Figure 11:**
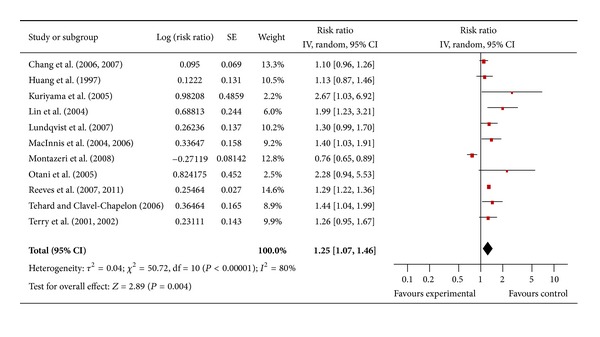
Obesity and postmenopausal breast cancer.

Eleven studies assessed the association between obesity and postmenopausal breast cancer (Figure 11). The pooled risk ratio demonstrated that obese women had an increased risk of postmenopausal breast cancer compared to women of normal weight (RR, 1.25; 95% confidence interval 1.07 to 1.46). There was significant heterogeneity across studies (heterogeneity *P* = 0.0001).

#### 3.4.11. Rectal Cancer

**Figure 12:**
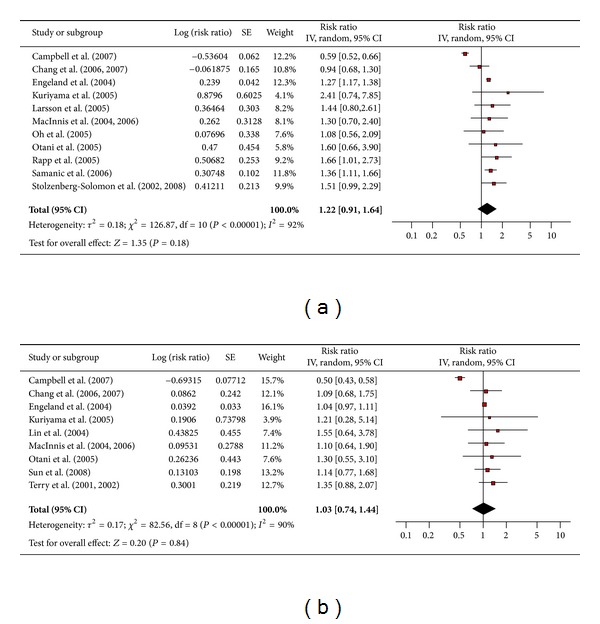
(a) Obesity and rectal cancer in men. (b) Obesity and rectal cancer in women.

There were 11 studies for men (Figure 12(a)) and nine for women (Figure 12(b)) that assessed the association between obesity and rectal cancer. The pooled risk ratio illustrated that obese men had no increased risk of rectal cancer compared to men of normal weight (RR 1.22; 95% confidence interval 0.91 to 1.64). There was significant heterogeneity across studies (heterogeneity *P* = 0.00001). Similarly among obese women the pooled risk ratio illustrated no increased risk of rectal cancer compared to women of normal weight (RR 1.03; 95% confidence interval 0.74 to 1.44). There was significant heterogeneity across studies (heterogeneity *P* = 0.00001). 

#### 3.4.12. Renal Cancer

**Figure 13:**
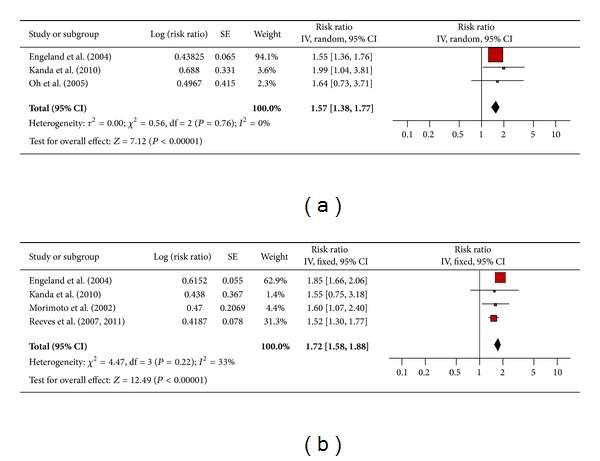
(a) Obesity and renal cancer in men. (b) Obesity and renal cancer in women.

There were 3 studies each for men (Figure 13(a)) and women (Figure 13(b)) that assessed the association between obesity and renal cancer. The pooled risk ratio illustrated that obese men had an increased risk of renal cancer compared to men of normal weight (RR 1.57; 95% confidence interval 1.38 to 1.77). There was no significant heterogeneity across studies (*P* = 0.76). Similarly, among obese women the pooled risk ratio illustrated an increased risk of renal cancer compared to women of normal weight (RR 1.72; 95% confidence interval 1.58 to 1.88). There was no significant heterogeneity across studies (heterogeneity *P* = 0.22). 

#### 3.4.13. Thyroid Cancer

**Figure 14:**
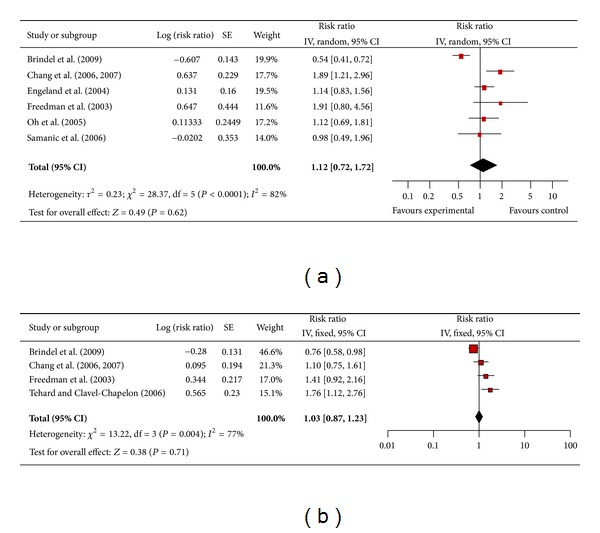
(a) Obesity and thyroid cancer in men. (b) Obesity and thyroid cancer in women.

There were 6 studies included in the meta-analysis assessing the association between obesity and thyroid cancer among men (Figure 14(a)) and 4 studies among women (Figure 14(b)). The pooled risk ratio illustrated that obese men had no increased risk of thyroid cancer compared to men of normal weight (RR 1.12; 95% confidence interval 0.72 to 1.72). There was significant heterogeneity across studies (*P* < 0.00001). Likewise, among obese women the pooled risk ratio demonstrated no increased risk of thyroid cancer compared to women of normal weight (RR 1.03; 95% confidence interval 0.87 to 1.23). There was significant heterogeneity across studies (*P* < 0.004). 

#### 3.4.14. Results Summary

Table 1 summarizes the results of the observed associations between obesity and the 13 cancers included in this meta-analysis. Among men a statistically significant increased risk of cancer was observed for the following 5 cancers: colon, gallbladder, malignant melanoma, pancreatic, and renal cancer. In addition, obese men had significantly lower risk of multiple myeloma in comparison to men of normal weight. Among women a statistically significant increased risk of cancer was observed for the following 8 cancers: colon, endometrial, esophageal, gallbladder, leukemia, pancreatic, postmenopausal breast, and renal. Obese women also had significantly lower risk of non-Hodgkin lymphoma compared to women of normal weight. 

#### 3.4.15. Population-Attributable Risk

As an example to illustrate the use of meta-analysis results reported in this study, the PAR% for men and women in Canada was calculated on cancers for which a statistically significant association between obesity and cancer incidence was observed (Table 2). The population prevalence of obesity in Canada in 2002 was 22.9% in men and 23.2% in women. Among men PAR% ranged from a low of 5.6% to a high of 11.5%. The PAR% for obesity among men was greatest for colon and renal cancer (11.5%), gallbladder cancer (9.7%), pancreatic (7.6%), and malignant melanoma (5.6%). When the 95% confidence intervals for PAR% for men are considered the results are particularly noteworthy. For example for colon cancer PAR% is as low as 9.9% but at the upper limit of the confidence interval PAR% is 13.1%. Likewise, for renal cancer the 95% confidence interval for PAR% ranged from 8.0% to 15%, while for pancreatic cancer, it ranged from 1.6% to 17.5%, and 1.6% to 9.9% for malignant melanoma. 

Among women PAR% ranged from a low of 4.2% to a high of 19.4%. The PAR% for obesity among women was greatest for esophageal adenocarcinoma (19.4%), endometrial (16.5%), gallbladder (16%), and renal cancer (14.3). When the 95% confidence intervals for PAR% for women are considered the results are also noteworthy. For example, at the upper limit of the confidence interval, PAR% was as high as 37.2% for esophageal adenocarcinoma, 27.7% for endometrial cancer, and 25.8% for gallbladder cancer. However, at the lower limit the PAR% was as low as 0.9% for colon cancer, 1.6% for postmenopausal breast cancer, and 1.8% for malignant melanoma.

## 4. Discussion

The results of this meta-analysis demonstrate a significant association between obesity and some of the 13 cancers for men and many of the 13 cancers for women. Obesity was significantly and positively associated with 5 of the possible 11 cancers relevant to men including, colon, gallbladder, malignant melanoma, pancreatic, and renal. A significant association was not observed among obese men for esophageal adenocarcinoma, leukemia, multiple myeloma, non-Hodgkin lymphoma, rectal, and thyroid cancer. Among women obesity was significantly and positively associated with 8 of the 13 cancers including, colon, endometrial, esophageal adenocarcinoma, gallbladder, leukemia, pancreatic, postmenopausal breast, and renal cancer. A significant association was not observed among women for malignant melanoma, multiple myeloma, non-Hodgkin lymphoma, rectal cancer, and thyroid cancer. 

The magnitude of the associations between obesity and cancer incidence in men and women, including the 95% confidence intervals, is particularly noteworthy. For example, obese men have an increased risk of developing colon and renal (RR both 1.57), gallbladder (1.47), pancreatic cancer (1.36), and malignant melanoma (1.26) in comparison to nonobese men. When the 95% confidence intervals are considered the results become even more compelling. For example the risk ratio is as high as 1.77 for renal cancer, 1.73 for pancreatic cancer and 1.65 for colon cancer. Even at the low end of the 95% confidence interval, cancer risk is still noteworthy at 1.48 for colon cancer, and 1.38 for renal cancer. 

The results for obese women are equally concerning. Obese women are not only at higher risk for developing cancer at more sites than men, the risk associated with some of the cancers is significantly higher. For example, obese women have an increased risk for developing esophageal adenocarcinoma (RR 2.04), endometrial (1.85), gallbladder (1.82), and renal cancer (1.72). Where statistically significant associations are observed only one risk ratio is less than 20% (colon 1.19), while the remaining risk ratios range between 1.25 (postmenopausal breast), 1.32 (leukemia), and 1.34 (pancreatic). The 95% confidence intervals are also alarming. For example, at the upper limit of the confidence interval the risk ratio is as high as 3.55 for esophageal adenocarcinoma, 2.65 for endometrial, and 2.50 for gallbladder cancer. 

The results also clearly demonstrate that a significant proportion of cancer incidence could be avoided, in Canada, by reducing the percentage of obese adults. The population-attributable risk percent for obesity in Canada was highest in men for five cancers: colon, renal, gallbladder, pancreatic, and malignant melanoma. Significant reductions in obesity therefore would dramatically decrease the incidence of those cancers. For women the greatest benefits from obesity reduction and prevention efforts would be observed for esophageal adenocarcinoma, endometrial, gallbladder, renal, pancreatic, leukemia, postmenopausal breast, and colon cancer. Significant reductions in these cancers for men and women in Canada would result in decreased health care expenditures and improved quality of life for many men and women in Canada. 

When the results of this meta-analysis are compared to those of Renehan et al. [6], who reviewed similar but earlier literature on obesity and cancer, a number of important similarities and differences are observed. First, there were some differences in the associations found between the two meta-analyses for men. Renehan et al. reported a significant and positive association for the following five cancers that was not reported in this meta-analysis: leukemia, multiple myeloma, non-Hodgkin lymphoma, rectal, and thyroid cancer. Furthermore, Renehan et al. did not report a significant and positive association for gallbladder and pancreatic cancer whereas a significant and positive association was reported in this paper. 

There was greater similarity between the results of this paper and Renehan et al. for women. For example, there were only two cancers, multiple myeloma and thyroid cancer where Renehan et al. reported a positive and significant association with obesity for women, and this paper did not. There were no differences for the remaining eleven cancers on the relationship between obesity and cancer in women. 

The second important difference between the two meta-analyses concerns the observed magnitude of the association and the coinciding 95% confidence intervals. Generally the results of our meta-analysis demonstrated higher magnitude of associations and confidence intervals. The most notable differences occurred for four cancers among men: renal, colon, pancreatic, and gallbladder cancer. For example, we reported a pooled risk ratio and 95% confidence interval of 1.57 (1.38, 1.77) for renal cancer, while Renehan et al. reported 1.24 (1.15, 1.34). Likewise, for colon cancer, we reported 1.57 (1.48, 1.65), while Renehan et al. reported 1.24 (1.20, 1.28), and for pancreatic cancer, we reported 1.36 (1.07, 1.73) and Renehan et al. reported 1.07 (0.93, 1.23). Finally, for gallbladder cancer, we reported 1.47 (1.17, 1.85) while Renehan et al. reported 1.09 (0.99, 1.21). 

Similar differences existed for obese women with the greatest differences in the pooled risk ratios and 95% confidence intervals being for three cancers: endometrial, renal, and gallbladder. For these cancers the magnitude of associations we reported is larger than those reported by Renehan et al. For endometrial cancer we reported a risk ratio of 1.85 (1.30, 2.65), while Renehan et al. reported 1.59 (1.5, 1.68). For renal cancer we reported 1.72 (1.58, 1.88) while Renehan reported 1.34 (1.25, 1.43), and for gallbladder cancer we reported 1.82 (1.32, 2.50) and Renehan, 1.59 (1.02, 2.47). There was only one cancer where Renehan et al. reported a larger risk ratio than we did, which was for non-Hodgkin lymphoma. 

There are some plausible explanations for these differences. Likely the most significant factor contributing to the differences in the results of the two meta-analyses are the different approaches to the statistical methods used to aggregate the data across studies. The approach used by Renehan assessed the risk in developing cancer as BMI increased every 5 points, from the lowest BMI category up to the highest category. This strategy incorporates the risk of those who have a BMI slightly above normal, to above normal to those identified as obese. Given the risk of developing cancer at lower BMI levels is generally less than that of obese persons which provides some explanation why Renehan et al's results generally illustrate lower overall risk and more narrow confidence intervals. 

In this paper we compared the risk of developing cancer between those in the lowest BMI category (normal healthy weight) and those considered obese (BMI 30+) category. Our meta-analysis therefore did not incorporate into the pooled risk ratio and 95% confidence intervals, the risk associated with developing cancer among those with slightly above normal and above normal BMI. Given the primary objective of this paper was to assess the relationship between obesity and cancer incidence we believe this was the most appropriate statistical analysis to answer the research question. The question Renehan et al. answered was broader in that they were interested in assessing across the spectrum of BMI from normal to obese, the relationship between BMI and cancer risk. 

A second major difference between the two reviews was our exclusion of studies judged to be of weak methodological quality. The results of studies of weak methodological quality are less trustworthy. The exclusion of these studies likely contributed to the higher pooled risk ratios observed in this meta-analysis in comparison to Renehan et al. The combined effect of the different statistical approaches and the exclusion of studies of weak methodological quality likely explains much of the observed differences between the reviews. 

 Finally, it may be that studies published since the Renehan et al. meta-analysis, report higher relative risks than had been reported previously. Given several studies published since 2007 were added to this paper, this may further explain the higher pooled risk ratios and 95% confidence intervals observed in this paper. 

It is important to note that the results from some of the studies included in this paper may not be relevant to a Canadian context. For example, studies conducted in South East Asian countries may have limited applicability in Canada. However, a rigorous and systematic meta-analysis process should not limit study inclusion by the country in which the study was conducted. As a result a decision was made to be more rather than less inclusive. Furthermore, in most instances, studies conducted in countries quite dissimilar to Canada constituted relatively little overall weight in the point estimate, and therefore the impact of the results of these studies on the point estimates is relatively small. 

## 5. Conclusions

These findings have important implications for public health practice globally as well as Canada. First, the evidence reported here continues to establish the evidence base suggesting a statistically significant and positive association between obesity and cancer risk. In comparison to nonobese men and women, obese men and women have considerably increased risk of developing cancer. Women are at particularly high risk for as many as eight cancers, and the magnitude of the association is very high. Similarly among obese men, there is an increased risk for five cancers. Given obesity generally is preventable public health efforts to implement effective population wide programs to reduce and prevent obesity are needed. 

## Figures and Tables

**Table 1:** Summary of results of meta-analysis of associations between obesity and cancer risk in 57 studies from 18 countries, 1985–2011.

Cancer	Males	Females
	RR and 95% CI	RR and 95% CI
Colon	1.57 (1.48, 1.65)*	1.19 (1.04, 1.36)*
Endometrial	NA	1.85 (1.30, 2.65)*
Esophageal	1.23 (0.58, 2.60)	2.04 (1.18, 3.55)*
Gallbladder	1.47 (1.17, 1.85)*	1.82 (1.32, 2.50)*
Leukemia	1.16 (0.88, 1.52)	1.32 (1.08, 1.60)*
Malignant melanoma	1.26 (1.07, 1.48)*	0.95 (0.84, 1.07)
Multiple myeloma	0.58 (0.36, 0.93)*	1.20 (0.99, 1.45)
Non-Hodgkins lymphoma	1.09 (0.98, 1.21)	0.91 (0.86, 0.97)*
Pancreatic	1.36 (1.07, 1.73)*	1.34 (1.22, 1.46)*
Postmenopausal breast	NA	1.25 (1.07, 1.46)*
Rectal	1.22 (0.91, 1.64)	1.03 (0.74, 1.44)
Renal	1.57 (1.38, 1.77)*	1.72 (1.58, 1.88)*
Thyroid	1.12 (0.72, 1,72)	1.03 (0.87, 1.23)

*Statistically significant at *P* < 0.05

**Table 2:** Population-attributable risk percent in Canada for obesity and cancer.

Cancer	Males	Males	Females	Females
	RR and 95% CI	Population-attributable risk Percent	RR and 95% CI	Population-attributable risk Percent
Colon	1.57 (1.48, 1.65)*	11.5% (9.9, 13.1)*	1.19 (1.04, 1.36)*	4.2% (0.9, 7.7)
Endometrial	NA	NA	1.85 (1.3, 2.65)*	16.5% (6.5, 27.7)*
Esophageal	1.23 (0.58, 2.60)	—	2.04 (1.18, 3.55)*	19.4% (4.0, 37.2)*
Gallbladder	1.47 (1.17, 1.85)*	9.7% (3.7, 16.3)*	1.82 (1.32, 2.50)*	16% (6.9, 25.8)*
Leukemia	1.16 (0.88, 1.52)	—	1.32 (1.08, 1.60)*	6.9% (1.8, 12.2)*
Malignant melanoma	1.26 (1.07, 1.48)*	5.6% (1.6, 9.9)*	0.95 (0.84, 1.07)	—
Multiple myeloma	0.58 (0.36, 0.93)*	—	1.20 (0.99, 1.45)	—
Non-Hodgkin lymphoma	1.09 (0.98, 1.21)	—	0.91 (0.86, 0.97)*	
Pancreatic	1.36 (1.07, 1.73)*	7.6% (1.6, 17.5)*	1.34 (1.22, 1.46)*	7.3% (4.9, 9.6)*
Postmenopausal breast	NA	NA	1.25 (1.07, 1.46)*	5.5% (1.6, 9.6)*
Rectal	1.22 (0.91, 1.64)	—	1.03 (0.74, 1.44)	—
Renal	1.57 (1.38, 1.77)*	11.5% (8.0, 15.0)*	1.72 (1.58, 1.88)*	14.3% (12.0, 16.9)*
Thyroid	1.12 (0.72, 172)	—	1.03 (0.87, 1.23)	—

* Statistically significant at *P* < 0.05
